# A pilot study on parvovirus B19 infection in paediatric haematological malignancies

**Published:** 2011-04

**Authors:** Janak Kishore, Manodeep Sen, Ashutosh Kumar, Archana Kumar

**Affiliations:** Department of Microbiology, Sanjay Gandhi Post Graduate Institute of Medical Sciences, Lucknow, India; * *Department of Pediatrics, CSM Medical University, Lucknow, India*; ** *Department of Pathology, CSM Medical University, Lucknow, India*

**Keywords:** ALL, DNA, induction therapy, leukaemia, lymphoma, parvovirus B19

## Abstract

**Background & objectives:**

Leukaemia and lymphoma are common paediatric haematological malignancies acquiring human parvovirus B19 (B19) infection. In some studies anaemia has been found in children with acute lymphoblastic leukaemia (ALL) during maintenance therapy and rarely in lymphoma. We studied frequency of B19 infection and its implications in new onset acute leukaemia (mostly ALL) and lymphoma in children.

**Methods:**

Seventy serum samples from 35 children (age <12 yr, 29 males) newly diagnosed with haematological malignancies (on induction therapy) were collected together with 34 controls (solid tumours). Children were examined clinically and for anti-B19 IgM antibodies by quantitative ELISA and B19 DNA by PCR (VP1-VP2) and nested-PCR (VP1 unique). Bone marrow aspirates were examined histopathologically, whenever possible.

**Results:**

Of the 35 children, 22 had acute leukaemia while 13 had lymphoma. B19 infection was seen in six (17.1%) of 35 children (5 ALL, 1 NHL), two at diagnosis and four during follow up compared to none in the control. Among five B19 IgM positive ALL (n=18) children, two had B19 genome and two had giant pronormoblasts (lantern cells; but one lacked B19 DNA). Of the 70 serum samples tested, eight (11.4%) had anti-B19 IgM as two children had persistent B19 infection and one showed atypical maculopapular rashes (lower limbs) while 12 (34.3%) had anti-B19 IgG antibodies. B19 infected children had unexplained anaemia (80%), required more blood transfusions (6.6 ± 4.8 Units vs 3.0 ± 2.6 Units) besides induction chemotherapy was delayed (60%) and required longer duration of therapy (29.2 ± 20 vs 6.3 ± 7.8 days) (*P*<0.02). Five children (2 ALL, 2 AML, 1 NHL) died but none were infected with B19.

**Interpretation & conclusions:**

B19 infection should be considered in children with ALL as it frequently caused unexplained anaemia and delay in induction chemotherapy.

The common paediatric haematological malignancies include leukaemia (30%) and lymphoma (12%) while acute lymphoblastic leukaemia (ALL) accounts for one-fourth of all childhood cancers and three fourth (75%) of all newly diagnosed patients with leukaemia. The incidence of ALL is 3-4 cases per 100,000 children below 15 yr of age with a peak age group of 2-5 yr^1^. Acute myeloid leukaemia (AML) accounts for 15-20 per cent of leukaemia in children. Lymphomas are the third most common malignancy in children and adolescents after leukaemia and brain tumours. About 60 per cent are non-Hodgkin’s lymphoma (NHL) and 40 per cent are Hodgkin’s disease (HD) lymphomas but are uncommon below the age of 5 yr and incidence increases with age^1^.

Human parvovirus B19 is a single stranded DNA virus in the genus erythrovirus of the family *Parvoviridae*. B19 has a wide spectrum of clinical manifestations and diagnosis is mainly done by the detection of B19 specific IgM antibodies or B19 DNA^2^. Children with haematological malignancies are anaemic due to malignant infiltrations of bone marrow, cytotoxic drugs and further it may be complicated by infection with B19 virus. The effects of B19 infection have been studied mostly as sporadic cases usually in ALL patients during the course of maintenance chemotherapy causing acute/chronic anaemia, pure red-cell aplasia or pancytopenia^3^, and less frequently thrombocytopenia and neutropenia^3^–^6^. Paediatric patients with ALL receiving chemotherapy have also been reported to be at high risk for acquiring parvovirus B19 infections^7^. Since B19 has a great tropism for erythroid progenitor cells^8^, it frequently leads to anaemic conditions.

Only a couple of large series reports have described either on its potential to precipitate varying forms of cytopenia in patients prior to or at the diagnosis of ALL and may infrequently serve as a prodrome to ALL or a persistent B19 infection^9^,^10^. Role of B19 infections in patients with myeloid leukaemia and lymphoma have rarely been studied^11^–^14^. B19 infection has been postulated to be an opportunistic infection or may have a putative role in pathogenesis, with a few studies exploring possible role of B19 infection preceding, mimicking or precipitating ALL^15^. Most studies have found the frequency of B19 infection in 8 to 18 per cent of cases of ALL^7^,^10^,^16^. Difficulty in the detection of B19 infection is due to limited diagnostic facility mostly confined to research laboratory and due to presence of virus at cryptic sites rather than in serum at time of diagnosis and viral titres in the serum may be low^10^. Frequency of parvovirus B19 infections and its implications in newly diagnosed cases of paediatric haematological malignancies on induction chemotherapy is largely unknown. Hence, a study was designed to find the frequency of B19 infection and its implications in newly diagnosed children with haematological malignancies during induction therapy and follow up.

## Material and Methods

*Study subjects*: Thirty five children (age <12yr, 29 males; male : female ratio of 4.8 : 1) with newly diagnosed haematological malignancies enrolled consecutively in the Department of Pediatrics, CSM Medical University, Lucknow, from August 2004 to July 2005 were included in the study and followed up to one year as per clinical requirements. All old cases with haematological malignancies or cases already in maintenance therapy were excluded from the present study.

Laeukemia cases were diagnosed on the basis of conventional methods which included detailed clinical examination, haemogram and bone marrow biopsy in accordance with French-American-British co-operative group^17^ while lymphoma cases were diagnosed as per the criteria of Rappaport *et al*^18^. Treatment protocols followed were according to “Children Oncology Group (COG) supported by “National Cancer Institute (NCI)” USA. Informed written consent was taken from parents of each child and institutional ethical committee cleared the study protocal.

*Control population*: Thirty four age matched controls attending the Department of Pediatrics, CSM Medical University, Lucknow were also included in the study of which 30 had solid tumours (Wilms, neuroblastoma, retinoblastoma, Ewings sarcoma, *etc*.) *i.e.*, not-involving haematopoietic system were selected while the rest four had other infections.

*Sample collection*: Blood samples were collected aseptically from 35 children with haematological malignancies (3-4 ml during induction therapy and then at 2-3 months or as per clinical requirement during follow up) and once from controls while bone marrow aspirates were examined histopathologically wherever possible in the cases.

*Follow up*: During follow up the children were observed clinically for fever, fatigue, pallor, anorexia, myalgia and for haemoglobin levels, total and differential leucocyte count, blood film morphology and platelet count. In case of persistent or prolonged anaemia or delay in induction therapy or other complications blood samples were also collected for B19 testing. Prolonged unexplained anaemia was defined as anaemia of >2 wk duration with haemoglobin levels < 10 g/dl or sudden drop of haemoglobin level of 2.5 g/dl without a readily attributable aetiology. Thus a total of 70 serum samples were collected from 35 children and were stored at -70°C until tested.

*Serological and molecular diagnosis*: This was performed in the Department of Microbiology, Sanjay Gandhi Post Graduate Institute of Medical Sciences, Lucknow. B19 virus specific IgM and IgG antibodies in serum samples were determined using quantitative ELISA kits (IBL Hamburg, Germany). Serum was diluted 1 in 101 and ELISA tests were performed as per manufacturer’s instructions. In IgM assay the set of positive control sera with 0, 10, 50 and 100 units of antibodies/ml was put up while in IgG assay positive control sera had 0.6, 3, 15 and 100 IU/ml besides in each assay a negative control serum and a blank well were put up, and all serum samples were tested in duplicate. Absorbance values (optical density; OD) were read against the blank at 450 nm in an ELISA reader (Tecan Austria GmbH, Austria; model Sunrise). The mean OD values were calculated for each controls and test sera and the OD values of set of positive controls were plotted on a semi-log paper and test sera with an OD value equal to or more than 24 units/ml were regarded as positive for anti-B19 IgM antibodies while for anti-B19 IgG it was 3.5 IU/ml.

*B19 DNA extraction and amplifications*: DNA was extracted from serum samples by QIAamp Ultrasens virus kit (Qiagen, Germany) as per manufacturer’s instructions. B19 DNA were amplified by PCR using primers from VP1-VP2 common region and yielded amplicons with 372 bp on gel electrophoresis and ethidium bromide staining as described previously^19^. B19 DNA isolated from a case of erythema infectiosum was used as a positive control (kindly donated by Dr Y. Matsunaga, NIID, Tokyo, Japan). Similarly a nested-PCR^20^,^21^ was done using a set of primers from VP1 unique regions of B19 namely, primer B3 – TGT GTG TTG TGT GCA AC- (nt 2193-2209) and primer B4 – CAAACTTCCTTGAAAATG – (nt 3119-3235). One microliter of PCR products was then subjected to second round of amplification using internal primers B5 –CAA AAGCATGTGGAGTGAGG-(nt2229-2245) and B6-GTGCTG TCA GTA ACC TG TAC- (nt 3065-3082). On agarose gel electrophoresis and ethidium bromide staining PCR amplicons after 35 cycles (Perkin Elmer, GeneAmp 9600, USA) were of 372 bp while in nested-PCR amplicons of 942 bp in the first round and 853 bp after second round of amplification respectively, were considered as B19 DNA positive. Recent parvovirus B19 infection was diagnosed by the presence of serum IgM antibodies or detection of B19 DNA.

*Statistical analysis*: The relationship between parvo-virus B19 infection and clinicopathologic variables were evaluated by χ^2^ test, Fisher’s exact or “t” test.*P*<0.05 was considered statistically significant.

## Results

Among 35 children with newly diagnosed haematological malignancies, 22 were diagnosed to have leukaemia, of whom 18 had ALL and four had AML. Thirteen children were diagnosed with lymphoma, of whom, 8 had non-Hodgkin’s lymphoma (NHL) and 5 had Hodgkin’s disease (HD). Clinically, mean age of children with ALL, AML, Hodgkin’s lymphoma and non Hodgkin’s lymphoma were 5.7 ± 2.8, 10.7 ± 1.5, 7.6 ± 1.8 and 6.8 ± 3.5 yr, respectively. Most children (31%) were in the age group of 8-10 yr. The male : female ratio among ALL and AML children was 2.6:1 and 3:1 respectively; while all lymphoma cases were male. Majority of the children had a high tumour burden as indicated by the presence of hepato-splenomegaly and lymphadenopathy. Haematological characteristics observed in children were anaemia to a significant degree and was seen in children with ALL (mean Hb: 5.7 ± 2.1 g/dl) as compared to NHL and HD cases. Higher mean total leucocyte count and absolute neutrophil count was seen in ALL, mean platelet count was least in children with ALL (92 ± 76 × 10^9^/ l) and highest in HD cases (230 ± 16 10^9^/ l) while AML and NHL children had intermediate values but no significant bleeding manifestations were seen.

**Fig. 1:**
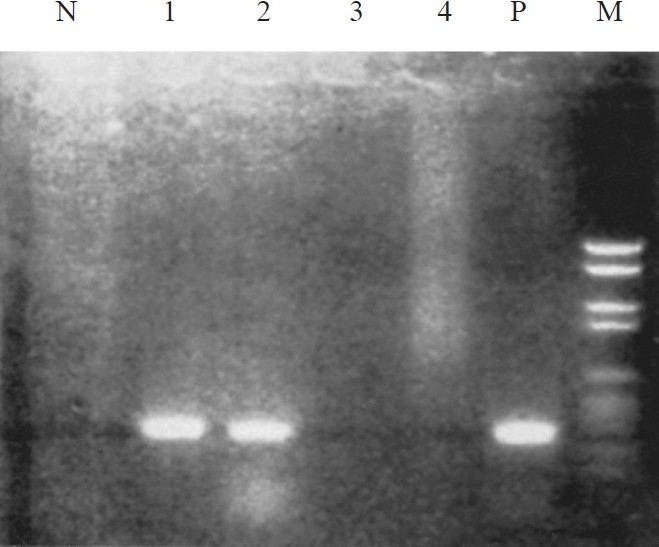
PCR for VP1-VP2 common region of B19 genome : agarose gel stained with ethidium bromide under ultra-violet illumination. Lane 1 to 4 patients serum samples; M, molecular weight marker; P, positive control (cloned B19 DNA) with 372 bp amplicons; N, negativ control.

**Fig. 2:**
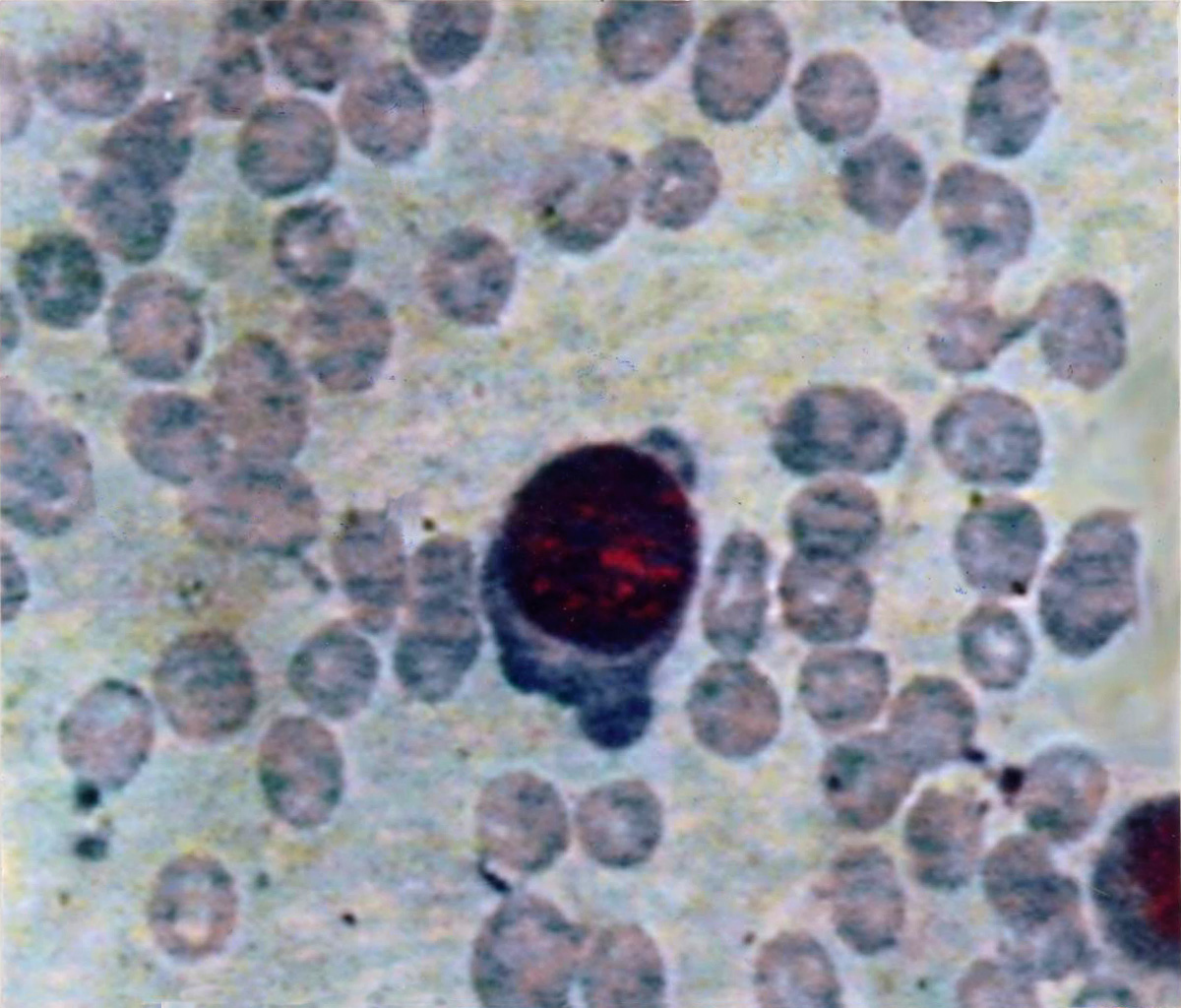
Giant pronormoblasts in Giemsa stained bone marrow smear in a case of ALL with dog’s ear projections and cytoplasmic vacuolization (20×100).

Recent parvovirus B19 infection as determined by the presence of serum IgM antibodies was found in 6 of 35 (17.1%) children with haematologic malignancies compared to none in controls (*P*<0.05). Thus, the relative risk of acquisition of B19 infection in children with haematologic malignancies was nearly 5.8 times compared to controls. Of the six B19 IgM positive children, five (83.3%) had ALL, one had NHL and all were males with three in younger age group (2-4 yr). Thus, among 18 children with ALL, five (27.8%) were infected with B19. Two children were B19 IgM positive at the time of first diagnosis while four children turned IgM positive during follow up and became negative on subsequent follow ups (Table I). Of the 70 serum samples collected from 35 children, eight had anti-B19 IgM from six cases (11.4%) since IgM persisted in two cases (case nos 2 & 4; Table I). B19 specific IgG was positive in 12 (34.3%) cases and in 4 (11.8%) controls. Anti-B19 IgM and IgG antibodies both were positive in three cases of ALL of whom B19 genome was also detected in two cases (case nos. 1 and 3) by both PCR (Fig. 1) and nested-PCR (data not shown) using primers derived from two different region of B19. One B19 IgM positive ALL child (case no.5), had continuous fever for 5 wk and developed atypical maculo-papular rashes on limbs during the follow up. On investigation, neutropenia was detected and bone marrow stained smear had giant pronormoblasts (lantern cell) (Fig. 2). Giant pronormoblasts were also seen in another case (case no. 3) of ALL who had both IgM and B19 DNA but no rash or arthropathy. Seasonally, among six anti-B19 IgM positive children four acquired B19 infection during late winter and early spring.

**Table I.** B19 antibodies by ELISA and B19 DNA by PCR in six B19 infected children

Serial No. (Patient No.)	No. of follow ups	B19 IgM antibodies	IgG antibodies	PCR (VP1-VP2 common region)	Nested- PCR VP1 unique region
1.	I	–	–	–	–
(Patient no. 2)	II	+	+	+	+
	III	–	+	–	–
	IV	–	+	–	–
	V	–	_	–	–
2.	I	++	–	–	–
(Patient no. 12)	II	+	++	–	–
	III	–	+	–	–
3.	I	–	–	–	–
(Patient no. 3)#	II	+	–	+	+
	III	–	+	–	–
4.	I	+	–	–	–
(Patient no. 15)*	II	++	–	–	–
	III	–	+	–	–
5.	I	–	–	–	–
(Patient no. 17)#	II	++	++	–	–
	III	–	–	–	–
6. (Patient no. 27)	I	–	–	–	–
	II	+	–	–	–
	III	–	+	–	–

S.No. 1-5 had ALL, S.No. 6 had NHL;

# Giant pronormoblast (Lantern cell) Seen;

* atypical maculopapular rash over both lower limbs

Out of 35 cases with leukaemia or lymphoma, seven were lost to follow up (3 of ALL, 4 of NHL) while five children (2 of ALL, 2 of AML and 1 of NHL) died and none of them were infected with B19. In the remaining 23 cases complete remission was achieved after induction therapy and they were followed up usually from 2 to 4 months and only a few for 9 months. However, adverse consequences were observed in these 23 children with haematological malignancies who got B19 infection (n=5) and on comparison with B19 uninfected (n=18) group it was observed that 4 of the 5 (80%) B19 infected children experienced prolonged unexplained anaemia (*P*<0.033) besides increased requirement for blood transfusion (*P*<0.05). The delay in induction chemotherapy was seen in 3 of 5 B19 infected children and they required much longer duration of induction therapy (*P*<0.02) (Table II).

**Table II.** Consequences in 23 children with haematological malignancies (5 IgM +ve, 18 IgM -ve)

S. No.	Clinical condition	Status of B19 IgM antibodies
1.	Prolonged unexplained anaemia	4 of 5 (80%) of IgM +ve group *vs* 4 of 18 (22 %) of IgM -ve group (*P*<0.033)
2.	Blood transfusion requirements	6.6 ± 4.8 Units in IgM +ve group *vs* 3.0 ± 2.6 Units in IgM -ve group (*P*<0.036)
3.	Delay in induction chemotherapy (> 7 days)	3 of 5 (60%) B19 IgM +ve group *vs* 5 of 18 (27.7 %) B19 IgM -ve group (*P*<0.18)
4.	Duration of delay in chemotherapy	29.2 ± 20 days in IgM +ve *vs* 6.3 ± 7.8 days in B19 IgM -ve (*P*<0.02)

## Discussion

The present pilot prospective hospital based study describes the clinical and haematological implications of parvovirus B19 infection mostly in ALL and in a small number of lymphoma cases. The pathophysiological role of B19 infection in interference with erythropoesis is due to direct cytopathic effect mediated by VP2 protein of B19 which inhibits colony formation of blast forming units (BFU) in the bone marrow and immunological mediation by cytokines TNF-α and interferon-γ which may even result in pancytopenia^8^. Hence B19 infected children required frequent blood transfusions. Since the receptor for B19 is blood group ’P’ antigen (tetrahexose ceramide), it has great tropism for erythroid cell precursors in the bone marrow.

In the present study, the frequency of parvovirus B19 specific IgM antibodies positivity was found to be 17.1 per cent, anti-B19 IgG positivity was 34.3 per cent and B19 DNA in two (5.7%) cases. In one Egyptian^7^ study on ALL cases B19 IgM positivity was 26 per cent, IgG positivity was 38 per cent and 8 per cent had B19 DNA. In another Swedish study^16^ on 117 children with ALL during the maintenance treatment B19 DNA was found in 15 per cent cases with increased number of complications like cytopenia causing significantly longer periods of unwanted interruptions of chemotherapy besides higher number of blood transfusions. Within ALL we also had 11.1 per cent cases with B19 DNA which becomes comparable with these two studies.

In the present study, majority of cases turned positive during late winter and early spring which are known seasons of outbreak of B19 since environment conditions are conducive for virus transmission^2^. B19 IgM was found more commonly in the age group of 2-4 yr possibly due to their susceptibility to B19 infection or because of maximum number of ALL patients being in this age group. In Indian children B19 seroprevalence is 8.9 per cent in children of 1-5 yr age^22^.

In 2003, a study from London opined that erythroid suppression and immune cell proliferation were associated with B19 infection and might also be important in the pathogenesis of acute leukaemia as B19 DNA positivity was found in 21.4 per cent of ALL and 50 per cent of AML patients^10^. However, our study was not aimed to find the pathogenetic mechanism and DNA positivity was much lower which could be due to delays in patients’ reporting to the hospital by which time B19 DNA came down to undetectable levels.

In the present study, one B19 IgM positive patient developed features of erythema infectiosum in the form of atypical maculo-papular rashes on both the lower limbs which has seldom been reported^23^,^24^. The same patient also had giant pronormoblasts or “Lantern cell” in bone marrow aspirate and which is suggestive of B19 infection^25^.

Acute B19 infection causes intense viraemia, hence B19 DNA can be detected in the serum by PCR. Since DNA may circulate for a few days (7-12 days) only, it should be coupled with the detection of IgM antibodies which is a reliable indicator of a recent B19 infection and lasts for 2 to 3 months or longer^1^. Further detection of free DNA in serum denotes an active infection besides it is important in immunocompromised conditions like leukaemia where patients may fail to mount sufficient quantities of virus specific IgM antibodies but PCR can be positive. Hence, both of these techniques were employed to detect active B19 infection. In the present study, B19 DNA was detected in serum by PCR from VP1-VP2 common region as well as nested-PCR from VP1 unique region of B19 virus. Demonstration of DNA from two different regions of the virus genome further confirms the presence of B19 genome in the serum. The limit of detection in our PCR was found to be 2.4 × 10^2^ genome equivalents/ml of serum^26^ while sensitivity of nested-PCR is known to be 10-12 copies/ml. Thus our PCR positive cases had viral load higher than this value. Further, it has been reported that B19 infection can precede the clinical presentation as ALL even by five months^9^. Hence actual numbers of B19 infected children with ALL may be more than were actually detected.

In lymphomas, only a few studies are available on HD and NHL where anaemia or pure red cell aplasia similar to leukaemia cases has been observed. A study from Denmark reported a case of severe anaemia caused by B19 infection in a patient with autoimmune haemolytic anaemia and a B-cell NHL on detection sharply increased B19 viral load, reticulocytopenia and shortened erythrocyte life-span which are suggestive of a primary parvovirus infection^12^. In a study from Turkey parvovirus B19 infection was found in 3 of the 8 patients with Hodgkin’s lymphoma and in 5 of 10 patients with NHL^27^. In our study, of the 13 lymphoma cases, only 1 of the 8 NHL children and none of 5 HD cases, were infected with B19 and were amenable to treatment while four cases of NHL were lost to follow up.

Actual number of children infected with B19 virus may be more than observed in the present pilot study because of immunocompromised status causing failure to mount detectable quantities of IgM antibodies and brief DNAemia^2^. In patients with persistent B19 infection administration of human immunoglobulin (IVIG) has been recommended. [Our cases were not treated for B19 infection owing to lack of recognition of its clinical implications in cases of leukaemia and lymphoma by clinicians and due to high cost of treatment]. Our observation of mortality among children who were not B19 infected needs to be explored with further large scale prospective studies. The possible role of B19 virus in precipitating pre-ALL to ALL remains speculative. However, B19 should be considered in cases of unexplained anaemia or delay in induction therapy in children with haematologic malignancies. Further, the pathogenetic mechanisms causing delay in induction therapy are unknown and are areas for future research. To make an early diagnosis of B19 infection, a high degree of clinical suspicion is the only way as this infection is easily overlooked. Long-term longitudinal and multicentric large scale studies are required to determine the exact role of parvovirus B19 infection in paediatric haematological malignancies.
